# Triglyceride-glucose index in the development of peripheral artery disease: findings from the Atherosclerosis Risk in Communities (ARIC) Study

**DOI:** 10.1186/s12933-021-01319-1

**Published:** 2021-06-24

**Authors:** Jing-Wei Gao, Qing-Yun Hao, Ming Gao, Kun Zhang, Xiong-Zhi Li, Jing-Feng Wang, Dominique A. Vuitton, Shao-Ling Zhang, Pin-Ming Liu

**Affiliations:** 1 Department of Cardiology, Sun Yat-sen Memorial Hospital of Sun Yat-sen University, Guangzhou, 510120 China; 2 Department of Radiology, Sun Yat-sen Memorial Hospital of Sun Yat-sen University, Guangzhou, China; 3 EA3181, Université Bourgogne Franche-Comté, Besançon, France; 4 Department of Endocrinology, Sun Yat-sen Memorial Hospital of Sun Yat-sen University, Guangzhou, China

**Keywords:** Triglyceride-glucose index, Insulin resistance, Peripheral artery disease, Risk factors, Cardiovascular disease

## Abstract

**Background:**

It remains unclear whether triglyceride-glucose (TyG) index, a surrogate marker of insulin resistance, is prospectively associated with incident peripheral arterial disease (PAD).

**Methods:**

We included 12,320 Atherosclerosis Risk in Communities Study participants (aged 54.3 ± 5.7 years) free of a history of PAD at baseline (visit 1: 1987–1989). The TyG index was determined using ln (fasting triglycerides [mg/dL] × fasting glucose [mg/dL]/2), and measured at 5 visits between 1987 and 2013. Incident PAD was defined as the first hospitalization with PAD diagnosis or a new onset of measured ABI < 0.90 during follow-up visits. We quantified the association of both baseline and trajectories of TyG index with incident PAD using Cox regression and logistic regression analysis, respectively.

**Results:**

Over a median follow-up of 23 years, 1300 participants developed PAD. After adjustment for traditional PAD risk factors, each 1-SD (0.58) increase in TyG index was associated with an 11.9% higher risk of incident PAD [hazard ratio, 1.119 (95% CI, 1.049–1.195)]. Results were similar when individuals were categorized by TyG index quartiles [hazard ratio, 1.239 (95% CI, 1.028–1.492); comparing extreme quartiles]. Four distinct trajectories of stable TyG indexes at various levels along the follow-up duration were identified [low (22.2%), moderate (43.2%), high (27.5%), and very high (7.1%) trajectory groups]. Compared with those with a TyG index trajectory at a low level, those participants with TyG index trajectories at high and very high levels had an even greater risk of future incident PAD [odds ratio (95%CI): 1.404 (1.132–1.740) and 1.742 (1.294–2.344), respectively] after multivariate adjustments for traditional PAD risk factors.

**Conclusions:**

Higher TyG index is independently associated with an increased risk of incident PAD. Long-term trajectories of TyG index help identify individuals at a higher risk of PAD who deserve specific preventive and therapeutic approaches.

*Trial registration*: Clinical trial registration number: The ARIC trial was registered at clinicaltrials.gov as NCT00005131.

**Supplementary Information:**

The online version contains supplementary material available at 10.1186/s12933-021-01319-1.

## Background

Peripheral artery disease (PAD) is an important manifestation of systemic atherosclerosis affecting an estimated over 200 million people worldwide [1, 2]. It is associated with significant cardiovascular morbidity and mortality, with a variable spectrum of symptoms from none to severe when patients present with claudication or critical limb ischemia [3]. Despite its high prevalence and well-described adverse outcomes, the pathobiology of PAD is incompletely understood. There is, therefore, the need to identify potential biomarkers that could predict the risk of PAD to facilitate diagnosis and timely intervention at early stages of atherosclerosis.

Insulin resistance (IR), a pathophysiological state characterized by the attenuated insulin sensitivity of peripheral tissues, is the key feature of metabolic syndrome and type 2 diabetes [4]; and it contributes significantly to the development of atherosclerotic cardiovascular disease [5]. However, the role of IR in PAD has been inadequately explored, compared with that of other atherogenic mechanisms such as inflammation [6–8]. Moreover, risk factors for PAD have not been as thoroughly investigated as those for coronary heart disease (CHD) [9]. The triglyceride-glucose (TyG) index, which is calculated using fasting triglycerides (TG) and fasting glucose, is a reliable measure of IR [10, 11]. Growing evidence has demonstrated that the TyG index is related to morbidity and mortality of cardiovascular disease in the general population, patients with and those without diabetes [12, 13]. This is possibly because elevated TyG index by itself contributes to systemic arterial atherosclerosis, including carotid atherosclerosis and coronary artery calcification, an established marker of subclinical atherosclerosis [14, 15]. Previous studies evaluating association of the TyG index with markers of PAD, incident PAD or their complexity are inherently limited by small sample sizes, a retrospective study or a cross-sectional analysis, and by the use of measured TyG index at a single time point [16–18]. So far long-term specific prospective studies on the relationship between PAD and the TyG index, and its trajectory derived from the multiple measurements over time have not been performed.

We hypothesized that dynamic changes in IR over decades might modify the development of PAD. Accordingly, we used the data from the Atherosclerosis Risk in Communities (ARIC) study to evaluate the association of the TyG index with PAD and to determine the influence of baseline TyG index and different trajectories of its change over 20 years on the development of PAD.

## Methods

### Study population

The ARIC Study is a prospective cohort study that enrolled 15,792 participants aged 45 to 64 years, recruited between 1987 to 1989 from 4 US communities (Forsyth County, North Carolina; Jackson, Mississippi; eight northern suburbs of Minneapolis, Minnesota; and Washington County, Maryland), aimed at investigating the natural history, etiology, and clinical manifestations of atherosclerotic disease in black and white men and women. Cohort exams were conducted at visit 1 (1987–1989), visit 2 (1990–1992), visit 3 (1993–1995), visit 4 (1996–1998), visit 5 (2011–2013) and visit 6 (2016–2017); other exams are ongoing. ﻿Details about the study design have been previously described [19]. Written informed consent was obtained from all ARIC participants, and the ARIC study was approved by the institutional review boards at each site.

**Fig. 1:**
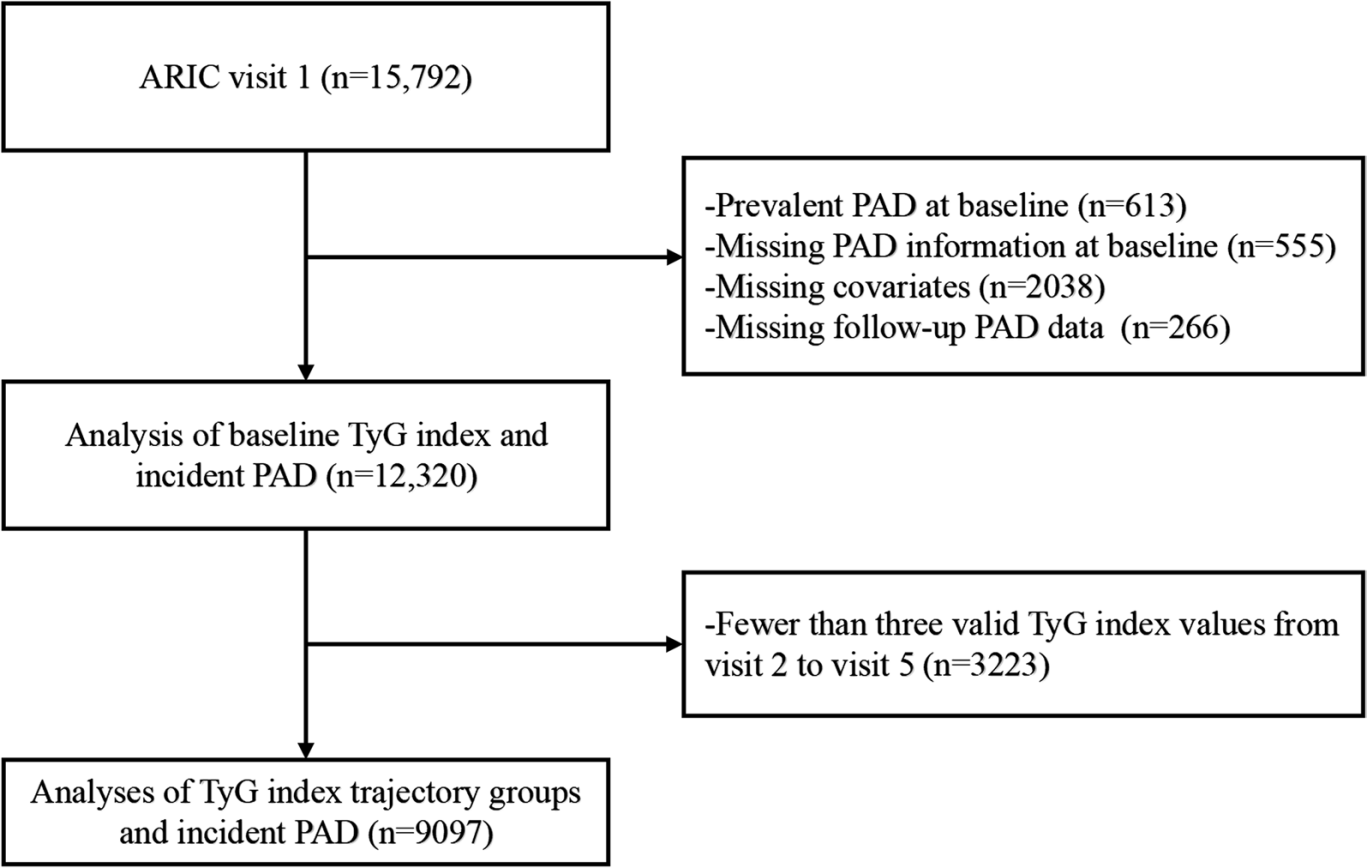
Flowchart for selecting the Atherosclerosis Risk in Communities Study participants for analysis

We excluded participants who had PAD diagnosis at baseline (n = 613); those who had missing data regarding PAD (n = 555); and those who had missing data regarding other covariates of interest (n = 2038). We also excluded participants who had no follow-up information on PAD (n = 266). This resulted in a final sample of 12,320 participants for the analysis of association between baseline TyG index and incident PAD. We further excluded those participants with fewer than three valid TyG index during follow-up visits; the remaining 9097 participants were included in the analysis of association between TyG index group-based trajectory and incident PAD (Fig. 1).

### Data collection at baseline

Trained interviewers collected information using standardized questionnaires on demographic, lifestyle, and detailed medical information at visit 1. Age, sex, race, educational level, physical activity, drinking and smoking status were self-reported. Specifically, physical activity was measured using the modified Baecke Physical Activity Questionnaire [20]. For each activity type, information related to the duration (hours per week) and frequency (number of weeks per month) were collected. Based on the Baecke Questionnaire scoring of summary estimates, index scores for sport, and leisure-time ranging from 1 to 5 (reflecting the highest activity level) were calculated. Leisure-time index score was based on the frequency of watching television, walking and bicycling (including to work), or shopping during leisure time whereas sport index score was based on the frequency of any sports and associated with sweat during leisure time [20]. Educational attainment was categorized as basic (less than high school), intermediate (high school graduate or vocational school), and advanced (college, graduate school, or professional school). Smoking and drinking status were classified as current, former, or never. Body mass index (BMI) was calculated as weight in kilograms divided by height in meters squared. Seated blood pressure represented the mean of the last two of three measurements using a random-zero sphygmomanometer after a 5-min rest. ﻿Hypertension was defined as systolic blood pressure readings ≥ 140 mmHg or diastolic blood pressure readings ≥ 90 mmHg, or use of antihypertensive drugs in the previous two weeks. Diabetes was defined as a fasting glucose level ≥ 126 mg/dL (≥ 7.0 mmol/L), a non-fasting glucose level ≥ 200 mg/dL (≥ 11.1 mmol/L), self-reported physician diagnosis of diabetes, or any use of antidiabetic drugs. History of CHD and stroke were both determined according to participants’ self-report or relevant measurements [8]. Medications were determined through self-reported usage in the previous two weeks and inspection of medication containers that participants brought to the visit.

Four biomarkers of inflammation and coagulation were measured at visit 1: fibrinogen, von Willebrand factor, factor VIII activity, and white blood cell count, from blood stored at − 70 °C using standardized protocols that have been described previously [21]. Plasma total cholesterol (TC), high-density lipoprotein cholesterol (HDL-C), and TG were also measured using automated enzymatic procedures, and low-density lipoprotein cholesterol (LDL-C) was calculated using the Friedewald equation when the concentration of TG is < 400 mg/dL [22]. The estimated glomerular filtration rate (eGFR) was calculated using the Chronic Kidney Disease Epidemiology Collaboration creatinine equation [23]. The TyG index was calculated as ln(fasting TG [mg/dL] × fasting glucose [mg/dL]/2). The ankle‐brachial index (ABI), as a surrogate marker of PAD, was calculated as the average of 2 resting ankle systolic pressure readings divided by the average of 2 resting brachial systolic pressure readings.

### Ascertainment of incident PAD

Based on previous literature [8, 24, 25], PAD-related hospitalizations were identified by the following International Classification of Diseases Ninth Revision (ICD-9) discharge codes: peripheral vascular disease, unspecified (443.9); atherosclerosis of native arteries of the extremities, unspecified (440.20); atherosclerosis of native arteries of the extremities with intermittent claudication (440.21); atherosclerosis of native arteries of the extremities with rest pain (440.22); atherosclerosis of native arteries of the extremities with ulceration (440.23); atherosclerosis of native arteries of the extremities with gangrene (440.24); other atherosclerosis of native arteries of the extremities (440.29); atherosclerosis of bypass graft of the extremities (440.3); chronic total occlusion artery extremities (440.4); atherosclerosis of other specified arteries (440.8); coexisting leg amputation (84.11, 84.12, 84.15, 84.17); leg artery revascularization (38.18, 39.25, 39.29, 39.50); lower extremity ulcer and gangrene (707.1x). Incident PAD was defined as the first hospitalization with diagnosis of PAD or a new onset of measured ABI < 0.90 during follow-up visits [3]. Critical limb ischemia (CLI), the severe form of PAD, was based on the discharge codes (84.11, 84.12, 84.15, 84.17, 707.1x). The follow-up period was set as the time from visit 1 (baseline) to the incidence of PAD, or loss to follow-up, or September 30, 2015, whichever came first. September 2015 was chosen as the last point of follow-up to maintain diagnostic consistency since ICD codes switched from ICD-9 to ICD-10 in October 2015.

### Statistical analysis

Normally distributed continuous data were expressed as mean ± SD, and the non-normally distributed continuous data, otherwise, were expressed as the median (interquartile range). Categorical data were expressed as numbers (percentage). Differences among groups were evaluated using analysis of variance (ANOVA) or Kruskal–Wallis *h*-test when appropriate for the continuous variables, and the χ^2^ test for the categorical variables. Kaplan–Meier estimates were used to compute cumulative incidence of incident PAD by TyG index quartiles and the differences in estimates were compared using the log-rank procedure. Cox proportional hazards regression model was used to calculate hazard ratios and 95% CIs between TyG index and time to incident PAD. Three multivariate models with progressive degrees of adjustment were used to adjust for potential confounders of PAD. Model 1 was adjusted for age, sex, and race. Model 2 was further adjusted for other clinical variables, including antihypertensive drugs, BMI, diabetes, drinking status, education level, sport and leisure time index scores, lipid-lowering drugs, SBP, and smoking status. Model 3 was additionally further adjusted for serum parameters, including eGFR, factor VIII activity, fibrinogen, LDL-C, von Willebrand factor, and white blood cell count. To determine whether our results were independent of the effects of potential drugs on TyG index, we also conducted a sensitivity analysis and repeated the aforementioned analyses by excluding those participants that were on any lipid- or glucose-lowing drugs. We further used a restricted cubic spline regression model with 3 knots to assess the nonlinear dose–response association between baseline TyG index and incident PAD. Subgroup analyses were performed stratifying by age, sex, race, smoking status, BMI, hypertension, and diabetes at baseline, respectively. Group-based trajectory analysis was designed to identify clusters of individuals with similar patterns of change over time [26]. We used latent class models to identify different patterns of longitudinal TyG index levels within the ARIC Study participants who had at least 3 TyG index measurements during follow-up visits. Models were fit using R 3.6.1 based on R package tidyLPA. We tested models with groups ranging from 2 to 5 and examined different criteria including Bayesian information criteria to assess the optimal number of trajectories so that no group included less than 5% of participants (Additional file: Table S1). Participants were assigned to the trajectory group for which they had the greatest posterior predictive probability. All the final models classified participants into trajectory groups with good discrimination: the mean probability of final group membership was 0.89. To estimate the association of TyG index trajectory groups with incident PAD, trajectory group was included as an independent variable in a logistic regression model examining predictors incident PAD at follow-up.

All analyses were conducted in SPSS version 23 (SPSS, Inc, Chicago, Illinois). A two-sided *P* value of < 0.05 was considered statistically significant.

## Results

### Baseline characteristics according to quartiles of TyG index

The average age of all the participants was 54.3 ± 5.7 years, 5693 (46.2%) were men, 3131 (25.4%) were current smokers, 1378 (11.2%) were diabetic, and 5192 (42.1%) had hypertension. The mean TyG index was 8.7 ± 0.6 (Table 1). We categorized the included population into 4 groups based on the quartiles of baseline TyG index (Table 1). Participants with a higher TyG index were older, and more often male and white; they had higher levels of BMI, SBP, DBP, TC, LDL-C, TG, fasting glucose, fibrinogen, Von Willebrand factor, factor VIII activity and white blood cell count; they had lower levels of education, physical activity scores (leisure time and sport index scores), HDL-C, and eGFR; they were also less frequently current drinkers (all *P* < 0.001). Likewise, participants in a higher TyG index quartile had a higher prevalence of hypertension, diabetes, CHD, and stroke; and were more prone to take antihypertensive drugs, and lipid-lowering drugs (all *P* < 0.001).

**Table 1:** Baseline characteristics of study participants by quartiles of TyG index

Characteristics	Total (n = 12,320)	Quartile 1 (n = 3080)	Quartile 2 (n = 3080)	Quartile 3 (n = 3080)	Quartile 4 (n = 3080)	*P* value
TyG index	8.7 ± 0.6	8.0 ± 0.2	8.4 ± 0.1	8.8 ± 0.1	9.4 ± 0.4	< 0.001
Age, years	54.3 ± 5.7	53.1 ± 5.7	54.2 ± 5.7	54.7 ± 5.7	55.1 ± 5.7	< 0.001
Male, %	5693 (46.2%)	1151 (37.4%)	1326 (43.1%)	1542 (50.1%)	1674 (54.4%)	< 0.001
White, %	9401 (76.3%)	2166 (70.3%)	2329 (75.6%)	2449 (79.5%)	2457 (79.8%)	< 0.001
BMI, kg/m^2^	27.6 ± 5.2	25.6 ± 4.8	27.0 ± 5.1	28.1 ± 5.1	29.7 ± 5.1	< 0.001
SBP, mmHg	120.8 ± 18.6	117.3 ± 18.9	119.3 ± 18.4	121.4 ± 17.9	125.2 ± 18.4	< 0.001
DBP, mmHg	73.4 ± 11.2	72.3 ± 11.5	72.9 ± 11.2	73.7 ± 10.9	74.9 ± 10.9	< 0.001
Smoking status, %						< 0.001
Current smoker	3131 (25.4%)	694 (22.5%)	823 (26.7%)	835 (27.1%)	779 (25.3%)	
Former smoker	4086 (33.2%)	918 (29.8%)	976 (31.7%)	1042 (33.8%)	1150 (37.3%)	
Never smoker	5103 (41.4%)	1468 (47.7%)	1281 (41.6%)	1203 (39.1%)	1151 (37.4%)	
Drinking status, %						< 0.001
Current drinker	7079 (57.5%)	1849 (60.0%)	1780 (57.8%)	1741 (56.5%)	1709 (55.5%)	
Former drinker	2304(18.7%)	488 (15.8%)	556 (18.1%)	613 (19.9%)	647 (21.0%)	
Never drinker	2937 (23.8%)	743 (24.1%)	744 (24.2%)	726 (23.6%)	724 (23.5%)	
Education level, %						< 0.001
Basic education	2756 (22.4%)	580 (18.8%)	678 (22.0%)	682 (22.1%)	816 (26.5%)	
Intermediate education	5077 (41.2%)	1201 (39.0%)	1239 (40.2%)	1328 (43.1%)	1309 (42.5%)	
Advanced education	4487 (36.4%)	1299 (42.2%)	1163 (37.8%)	1070 (34.7%)	955 (31.0%)	
Physical activity score						
Sport index score	2.5 ± 0.8	2.5 ± 0.8	2.5 ± 0.8	2.5 ± 0.8	2.4 ± 0.8	0.001
Leisure time index score	2.4 ± 0.6	2.4 ± 0.6	2.4 ± 0.6	2.4 ± 0.5	2.3 ± 0.6	< 0.001
Hypertension, %	5192 (42.1%)	979 (31.1%)	1170 (37.2%)	1370 (43.6%)	1779 (56.6%)	< 0.001
Diabetes, %	1378 (11.2%)	57 (1.8%)	129 (4.1%)	232 (7.4%)	984 (31.3%)	< 0.001
CHD, %	603 (4.9%)	67 (2.1%)	122 (3.9%)	175 (5.6%)	247 (7.9%)	< 0.001
Stroke, %	348 (2.8%)	66 (2.1%)	67 (2.1%)	96 (3.1%)	123 (3.9%)	< 0.001
Antihypertensive medication, %	3651 (29.6%)	586 (18.6%)	800 (25.4%)	976 (31.1%)	1370 (43.6%)	< 0.001
Lipid-lowering medication, %	347 (2.8%)	32 (1.0%)	77 (2.4%)	102 (3.2%)	140 (4.5%)	< 0.001
Fasting glucose, mg/dL	107.7 ± 37.0	94.2 ± 8.6	98.9 ± 11.5	103.7 ± 17.7	133.8 ± 63.1	< 0.001
HDL-C, mg/dL	51.7 ± 16.9	62.9 ± 17.7	54.6 ± 15.6	47.8 ± 13.9	41.7 ± 12.3	< 0.001
LDL-C, mg/dL	137.4 ± 38.8	121.4 ± 34.1	136.3 ± 37.4	146.2 ± 38.1	145.5 ± 40.4	< 0.001
TC, mg/dL	214.2 ± 41.0	197.0 ± 36.0	209.8 ± 38.0	220.4 ± 39.4	229.4 ± 43.3	< 0.001
TG, mg/dL	125.4 ± 64.9	63.6 ± 12.4	94.7 ± 13.2	131.7 ± 20.9	211.2 ± 62.8	< 0.001
eGFR, mL/min/1.73m^2^	102.1 ± 15.4	105.6 ± 14.8	102.5 ± 14.5	100.9 ± 15.4	99.9 ± 16.5	< 0.001
Fibrinogen, mg/dL	302.0 ± 64.5	294.03 ± 63.4	302.0 ± 64.2	303.7 ± 64.5	308.3 ± 65.1	< 0.001
Von Willebrand factor, % of standard	117.5 ± 47.8	112.5 ± 45.7	115.9 ± 45.3	116.7 ± 46.8	124.8 ± 52.2	< 0.001
Factor VIII activity, % of standard	130.5 ± 38.1	125.0 ± 35.2	128.0 ± 35.9	130.0 ± 36.1	139.3 ± 43.2	< 0.001
White blood cell count, × 10^9^	6.1 ± 2.0	5.6 ± 1.8	6.0 ± 1.9	6.3 ± 2.3	6.6 ± 1.9	< 0.001
Incident PAD, %	1300 (10.6%)	222 (7.2%)	278 (9.0%)	359 (11.7%)	441 (14.3%)	< 0.001
Incident CLI, %	186 (1.5%)	22 (0.7%)	20 (0.6%)	41 (1.3%)	103 (3.3%)	< 0.001

Values are mean ± SD for normally distributed data and median and interquartile range for non-normally distributed data, or n (%)

*ABI* ankle-brachial index, *BMI* body mass index, *CHD* coronary heart disease*, CLI* critical limb ischemia, *DBP* diastolic blood pressure, *eGFR* estimated glomerular filtration rate, *HDL-C* high-density lipoprotein cholesterol, *LDL-C* low-density lipoprotein cholesterol, *PAD* peripheral artery disease, *SBP* systolic blood pressure, *TC* total cholesterol, *TG* triglycerides, *TyG* triglyceride-glucose

### Association between baseline TyG index and incident PAD

**Fig. 2:**
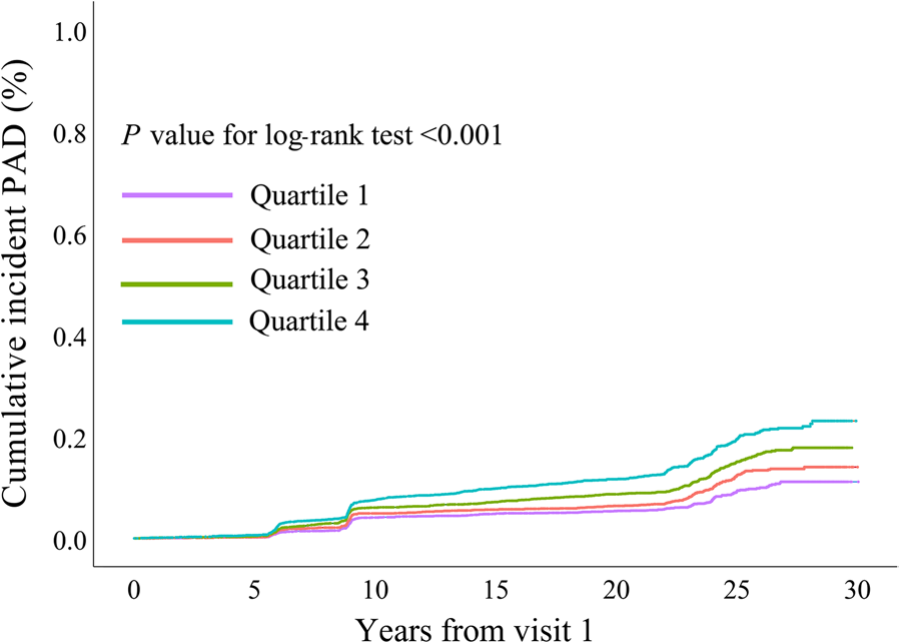
Cumulative incidence of incident PAD by quartiles of baseline TyG index. Cumulative incidence curves are statistically different (log-rank *P* < 0.001)

**Fig. 3:**
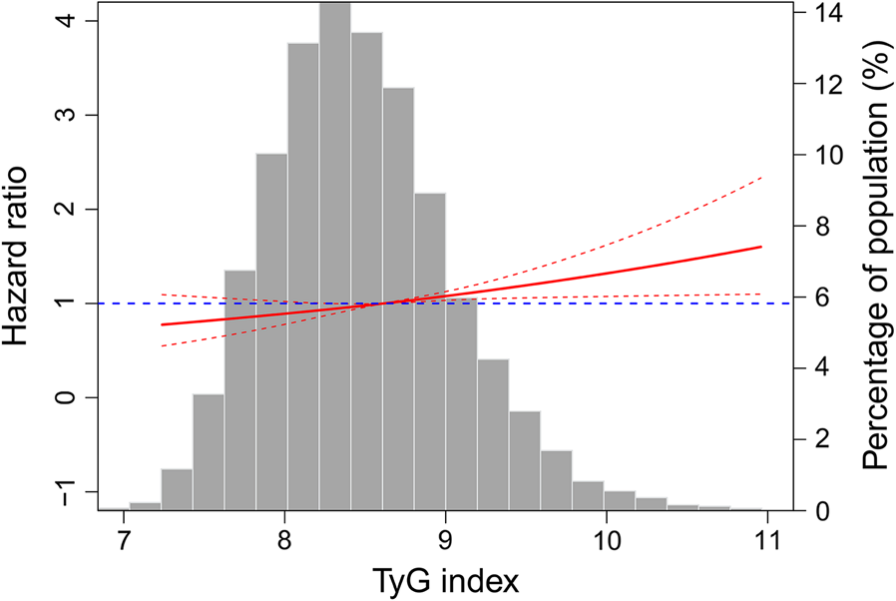
Adjusted hazard ratios of incident peripheral artery disease (PAD) by baseline triglyceride-glucose (TyG) index. Each hazard ratio was computed with a TyG index level of 8.6 as the reference. The hazard ratio was adjusted for age, antihypertensive medication, body mass index, diabetes, drinking status, education level, estimated glomerular filtration rate, factor VIII activity, fibrinogen, leisure time and sport index scores, lipid-lowering medication, low-density lipoprotein cholesterol, race, sex, smoking status, systolic blood pressure, von Willebrand factor, and white blood cell count. Red solid line represents the hazard ratio of TyG index across the whole range. Red dotted lines represent the 95% CI. Blue dotted line is the reference line as hazard ratio = 1. Histograms represent the frequency distribution of baseline TyG index

During a follow-up of 23.0 (14.7, 25.5) years, 1300 incident cases (10.6%) of PAD were observed. As Table 1 shows, the risk of incident PAD increased with increasing quartiles of TyG index [quartiles 1–4: 222 (7.2%) *vs.* 278 (9.0%) *vs.* 359 (11.7%) *vs.* 441 (14.3%); *P* < 0.001]. A similar trend in incident CLI was observed. In the multivariate model that measured TyG index as a continuous variable, a 1-SD increase (corresponding to 0.58) in TyG index was associated with an 11.9% higher risk of incident PAD after full adjustment for the potential confounders [hazard ratio, 1.119 (95% CI, 1.049–1.195); *P* = 0.001; Table 2]. Results were similar when we categorized individuals by TyG index quartiles: ﻿the highest risk of incident PAD was observed in the participants with the highest TyG index quartile, in 3 different adjusted models (all  *P* < 0.05, Table 2). In the final model, the hazard ratios (95%CIs) for incident PAD comparing the second, third, fourth quartiles of TyG index with the first quartile were 1.040 (95% CI, 0.869–1.245), 1.208 (95% CI, 1.013–1.441), and 1.239 (95% CI, 1.028–1.492), respectively (model 3 in Table 2; Fig. 2). ﻿These findings were not modified when the analysis included only participants without any lipid- or glucose-lowering medication (Additional file 1: Tables S2). Figure 3 shows the restricted cubic splines of the risk of incident PAD across levels of TyG index. Consistent with the analysis using quartiles of sample distribution, the risk of incident PAD increased in participants with a higher TyG index. However, there was no significant difference for the risk of incident PAD in participants with TyG index < 8.6 (Fig. 3).

**Table 2:** Risk of incident PAD for baseline TyG index

TyG index	Events/No. at risk	Model 1 HR (95% CI)	*P* value	Model 2 HR (95% CI)	*P* value	Model 3 HR (95% CI)	*P* value
Quartile 1	222/3080	Reference	1.0	Reference	1.0	Reference	1.0
Quartile 2	278/3080	1.290 (1.081–1.538)	0.005	1.097 (0.918–1.312)	0.307	1.040 (0.869–1.245)	0.666
Quartile 3	359/3080	1.671 (1.413–1.975)	< 0.001	1.301 (1.094–1.546)	0.003	1.208 (1.013–1.441)	0.035
Quartile 4	441/3080	2.232 (1.900–2.623)	< 0.001	1.339 (1.116–1.606)	0.002	1.239 (1.028–1.492)	0.024
Per 1 SD (0.58)	1300/12320	1.403 (1.332–1.478)	< 0.001	1.148 (1.078–1.222)	< 0.001	1.119 (1.049–1.195)	0.001

Model 1: Adjusted for baseline age, race, and sex

Model 2: Adjusted for model 1 covariates plus baseline antihypertensive medication, body mass index, diabetes, drinking status, education level, leisure time and sport index scores, lipid-lowering medication, systolic blood pressure, and smoking status

Model 3: Adjusted for model 2 covariates plus baseline estimated glomerular filtration rate, factor VIII activity, fibrinogen, low-density lipoprotein cholesterol, von Willebrand factor, and white blood cell count

*CI* confidence interval, *HR* hazard ratio, *PAD* peripheral artery disease, *TyG* triglyceride-glucose

**Fig. 4:**
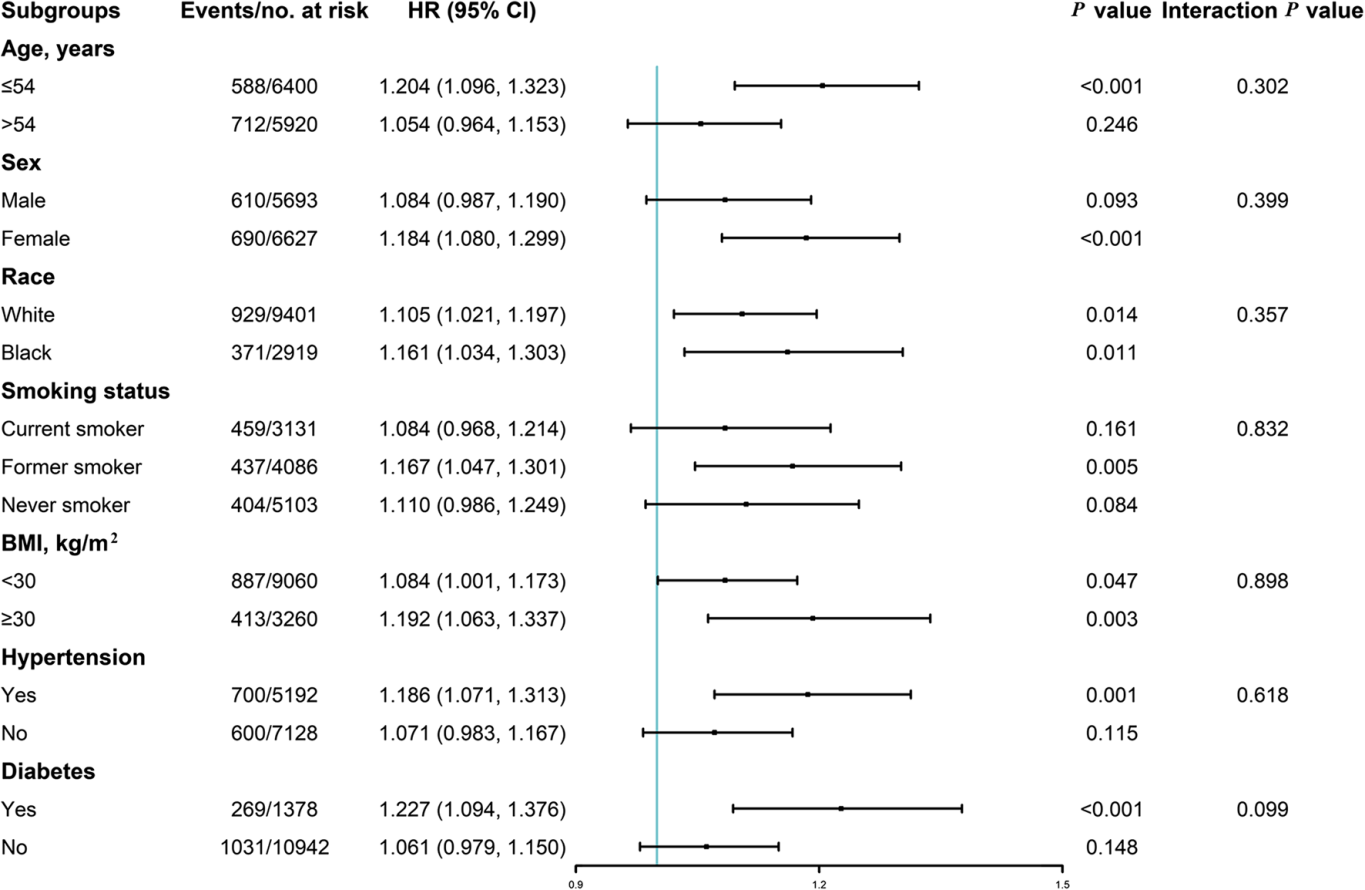
Subgroup analysis of the association between baseline TyG index and incident PAD. Cox regression after adjustment for antihypertensive medication, drinking status, education level, estimated glomerular filtration, factor VIII activity, fibrinogen, leisure time and sport index scores, lipid-lowering medication, low-density lipoprotein cholesterol, systolic blood pressure, von Willebrand factor, and white blood cell count was performed in subgroups according to age (≤ 54 or > 54 years), gender (male or female), race (White or black), smoking status (current or former or never), body mass index (BMI; < 30 or ≥ 30 kg/m^2^), hypertension (yes or no), and diabetes (yes or no)

When participants were stratified by age (≤ 54 or > 54 years), sex (male or female), race (white or black), smoking status (current, former or never), body mass index (< 30 or ≥ 30 kg/m^2^), hypertension (yes or no), and diabetes (yes or no), the association between TyG index and incident PAD remained consistent (all *P* for interactions > 0.05; Fig. 4).

### Association between TyG index trajectories and incident PAD

**Fig. 5:**
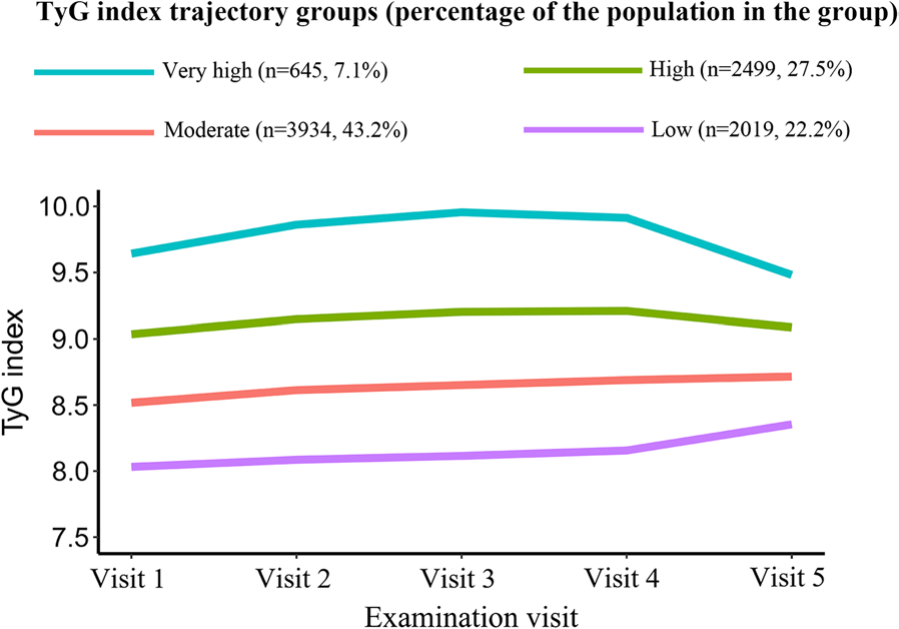
Trajectories by TyG index in the Atherosclerosis Risk in Communities Study

**Fig. 6:**
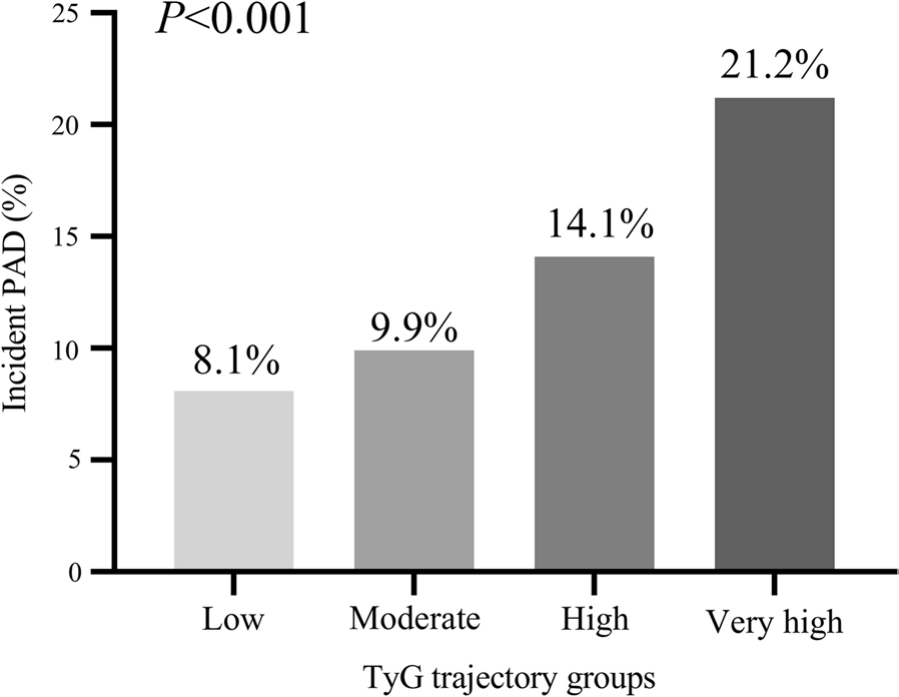
Prevalence of incident PAD across the TyG index trajectory groups

A total of 9097 participants were included for further trajectory analysis (Fig. 1). Four discrete trajectories with stable TyG indexes at various levels from visit 1 to visit 5 were identified (Fig. 5): low (n = 2019, 22.2%), moderate (n = 3934, 43.2%), high (n = 2499, 27.5%), and very high (n = 645, 7.1%) TyG index trajectory groups. The median (interquartile range) changes in TyG index level during the visits for these trajectory groups were 0.06 (− 0.02 to 0.14) in the low trajectory group, 0.05 (− 0.04 to 0.14) in the moderate trajectory group, 0.03 (− 0.07 to 0.14) in the high trajectory group, 0.05 (− 0.11 to 0.18) in the very high trajectory group (Additional file 1: Tables S3). As shown in Fig. 6, the rates of incident PAD were 8.1%, 9.9%, 14.1%, 21.2%, in the low, moderate, high and very high. TyG index trajectory groups, respectively (*P* < 0.001). Multivariate logistic regression analyses identified those with TyG index trajectory at high and very high levels as having an even greater risk of incident PAD in 3 different adjusted models (all *P* < 0.05, Table 3). In the fully adjusted model, compared with those with a low trajectory at a low level, the odds ratios (95% CIs) for associations of those participants with TyG index trajectories at the moderate, high, and very high levels with the risk of incident PAD were 1.063 (95% CI, 0.871–1.298; *P* = 0.547), 1.404 (95% CI, 1.132–1.740; *P* = 0.002), and 1.742 (95% CI, 1.294–2.344; *P* < 0.001), respectively (model 3 in Table 3).

**Table 3:** Risk of incident PAD for various levels of TyG index trajectory groups

TyG index trajectories	Model 1 OR (95% CI)	*P* value	Model 2 OR (95% CI)	*P* value	Model 3 OR (95% CI)	*P* value
Low	Reference	1.0	Reference	1.0	Reference	1.0
Moderate	1.279 (1.054–1.551)	0.012	1.143 (0.938–1.392)	0.184	1.063 (0.871–1.298)	0.547
High	1.957 (1.604–2.387)	< 0.001	1.552 (1.258–1.915)	< 0.001	1.404 (1.132–1.740)	0.002
Very high	3.132 (2.440–4.022)	< 0.001	1.932 (1.441–2.590)	< 0.001	1.742 (1.294–2.344)	< 0.001

Model 1: Adjusted for baseline age, race, and sex

Model 2: Adjusted for model 1 covariates plus baseline antihypertensive medication, body mass index, diabetes, drinking status, education level, leisure time and sport index scores, lipid-lowering medication, systolic blood pressure, and smoking status

Model 3: Adjusted for model 2 covariates plus baseline estimated glomerular filtration rate, factor VIII activity, fibrinogen, low-density lipoprotein cholesterol, von Willebrand factor, and white blood cell count

*CI* confidence interval, *OR* odds ratio, *HR* hazard ratio, *PAD* peripheral artery disease, *TyG* triglyceride-glucose

## Discussion

In this large-scale, community-based prospective cohort of middle-aged adults, we show for the first time that higher levels of TyG index are significantly associated with an increased risk of PAD over a median follow-up of 23 years. Furthermore, we identify that the 4 distinct trajectories of TyG index confer different risk of PAD, and a two-decade trajectory with elevated TyG index carries a greater risk of future incident PAD. These findings suggest a potential role for long-lasting high level of IR in the pathogenesis of PAD.

IR has been considered as an important risk factor for cardiovascular disease [27, 28]. Methods to directly measure IR are invasive, complex, and costly [10, 11]. Therefore, a number of surrogate markers of IR have been proposed and compared with the gold standard of the hyperinsulinemic-euglycemic clamp [29]. Homeostatic model assessment of IR (HOMA-IR), which is calculated by fasting insulin and glucose, is commonly used for assessing IR. However, the insulin concentrations are not routinely measured in clinical settings. As for the ARIC cohort, there were many missing insulin values due to low detection rate and the absence of insulin measurement at visit 2 and visit 3. Therefore, in this large-scale, community-based prospective cohort study, we utilized the TyG index as a biomarker of IR. The TyG index has been proved to be highly correlated with the euglycemic-hyperinsulinemic clamp test [10], and thus has a validity similar to HOMA-IR [11]. The immense advantage of using such a simple method of IR identification is obviously that it is easily accessible in any clinical settings, making our findings immediately usable by clinicians.

Among the multiple pathological consequences of atherosclerosis, PAD has generally been paid far less attention than CHD or stroke. Based on the updated estimates of PAD prevalence at global regional levels in 2015, 236.62 million (5.56%) people aged 25 years and older had PAD, among whom 73% were in low-income and middle-income countries [30]. However, only about 10% of patients with PAD demonstrate the typical symptomatology of intermittent claudication; and the majority of patients with PAD are thus asymptomatic and underdiagnosed [31]. Therefore, it is of great importance to regularly measure markers of risk for PAD and take preventive measures at an earlier clinical stage. In addition to age, significant atherosclerotic risk factors for PAD include cigarette smoking, dyslipidemia, and diabetes [32]. It has been well-established that IR and coexisting hyperinsulinemia are implicated in the development of dyslipidemia, hypertension, hypercoagulability, and atherosclerosis [33, 34]. However, there is a paucity of prospective data regarding the association between IR assessed by HOMA-IR and PAD [35, 36]. A cross-sectional study of 3242 adults from data in the National Health and Nutrition Examination Survey has identified a positive association between IR and PAD [35]. Only one community-based longitudinal study, which enrolled 4208 participants over the age of 65 years in the Cardiovascular Health Study, showed that IR was associated with a higher risk of clinical PAD [36]. In line with previous studies, our study of a larger sample size showed that the metabolic risk factors such as hypertension, diabetes, and hyperlipidemia, were more obvious among participants of higher quartiles of TyG index. Meanwhile, individuals with the highest quartile of baseline TyG index had a 2.23-fold higher risk for developing PAD than those with the lowest quartile. The association remained statistically significant after adjusting for all the aforementioned PAD risk factors. These findings suggested that the clinical management of TyG index may bring additional effect on PAD development even under vigorous control of traditional risk factors. Further studies are needed to unravel this aspect. Most previous studies based on the TyG index measured at a single time point [12, 13, 16–18], which may not reflect long-term exposure, for the TyG index levels may vary over time. Therefore, measurements of long-term trajectories of TyG index provide more reliable and robust results. Our study is the first, to our knowledge, to investigate the impact of long-lasting IR at various levels assessed by TyG index on future PAD incidence. We highlighted the fact that within the ARIC population there were heterogeneous patterns of trends in TyG index, which cannot be fully identified by the baseline TyG index levels and that such risk groups may change during follow-up. Different from the initial TyG index levels, long-term TyG index trajectory actually reflects the chronic impact of TyG index on incident PAD. Our results further suggest that those trajectory groups with long-term high and very high TyG index levels beginning in midlife are at a greater risk of incident PAD over 20 years after adjustment for traditional PAD risk factors. The participant’s electronic medical record allows a rapid integration of data across multiple time points. Thus, longitudinal measurements and recording of TyG indexes to identify TyG index trajectories are feasible and it represents an added value to the baseline levels to plan and monitor participants’ follow-up. In clinical practice, we can graph trends in TyG index to identify high-risk individuals who behave similarly to those with TyG index trajectories at high and very high levels (both mean TyG index > 8.6) that were observed in the present analysis. Such population may benefit from earlier and more frequent screening for PAD and aggressive risk factor management (e.g., blood pressure control, smoking cessation, maintaining metabolic health, etc.).

Our findings at least in part support the important role of impaired systemic glucolipid metabolism in the pathophysiology of PAD [37]; higher TyG index is a surrogate marker of IR, which is responsible for chronic hyperglycemia and also dyslipidemia including high plasma TG [38, 39]. However, many studies have indicated that IR could promote atherosclerosis not only through mechanisms that involve systemic factors, such as dyslipidemia, hypertension, and a proinflammatory state, but also through the effect of perturbed insulin signaling at the level of the intimal cells [34, 40]. Therefore, further studies are warranted to elucidate the precise mechanism for the observed association.

## Study limitations

Several limitations of this investigation are worth noting. The study included only whites and blacks aged 45–64 years at baseline, results may differ outside this age range and in other ethnicities. Due to the missing records of insulin levels in the ARIC study, we cannot compare trajectories of TyG index with HOMA-IR for predicting incident PAD. Inflammation-induced endothelial dysfunction was demonstrated to be at least one possible biological pathway between IR and atherosclerosis [41]. High-sensitivity C-reactive protein was not collected at baseline in the ARIC database and thus we unfortunately could not study the possible association with this sensitive marker of systemic inflammation [8, 42, 44]. However, we could analyze other inflammation biomarkers-albeit less sensitive-(fibrinogen and white blood cell count) as well as coagulation factors (von Willebrand factor, factor VIII activity) that were available from the ARIC database [42], as studies also support the role of these hemostatic factors in PAD [37, 43]. Moreover, because of the nature of any observational studies, we cannot exclude the possibility of residual confounders despite our careful adjustment for the well-known and suspected risk factors.

## Conclusions

Higher TyG index is associated with incident PAD, which is independent of other traditional atherosclerotic risk factors; suggesting that IR is actually involved in the pathogenesis of PAD. Trajectories denoting long-term exposure to high IR assessed by TyG index provide additional information about the cumulative burden of risk for future PAD. The results support the contribution of higher TyG index to the development of PAD and have implications on its prevention and treatment.

## Supplementary Information

**Additional file 1**: **Table S1**. Group-based trajectory model fit summary (N=9097). **Table S2**. Risk of incident PAD for baseline TyG index among participants without any lipid- or glucose-lowering medication. **Table S3**. TyG index at examination visits by trajectory groups of TyG index.

## Data Availability

The data that support the findings of this study are available from the corresponding author upon reasonable request.

## Abbreviations

ABI: Ankle‐brachial index
ARIC: Atherosclerosis Risk in Communities Study
BMI: Body mass index
CHD: Coronary heart disease
CLI: Critical limb ischemia
DBP: Diastolic blood pressure
eGFR: Estimated glomerular filtration rate
HDL-C: High-density lipoprotein cholesterol
HOMA-IR: Homeostatic model assessment of insulin resistance
IR: Insulin resistance
LDL-C: Low-density lipoprotein cholesterol
PAD: Peripheral artery disease
SBP: Systolic blood pressure
TC: Total cholesterol
TG: Triglycerides
TyG: Triglyceride-glucose

## Code availability

Data described in the manuscript, code book, and analytic code will be made available upon request pending application and approval. But we will make the data used in the manuscript, code book, and analytic code available to editors upon request either before or after publication for checking.
