# Supplementary material for: Triglyceride-glucose index in the development of peripheral artery disease: findings from the Atherosclerosis Risk in Communities (ARIC) Study
Source: Cardiovasc Diabetol. 2021 Jun 24;20:126. doi: 10.1186/s12933-021-01319-1 (PMC8223290; doi:10.1186/s12933-021-01319-1)
Supplement: Supplementary file 1 — Additional file 1: Table S1. Group-based trajectory model fit summary (N=9097). Table S2. Risk of incident PAD for baseline TyG index among participants without any lipid- or glucose-lowering medication. Table S3. TyG index at examination visits by trajectory groups of TyG index. [file 12933_2021_1319_MOESM1_ESM.docx]

**Table S1** Group-based trajectory model fit summary (N=9097).

| Model | AIC | BIC | SABIC | Latent class probabilities |
| --- | --- | --- | --- | --- |
| 2 | 62300.237 | 62414.088 | 62363.243 | 0.372/0.628 |
| 3 | 55647.643 | 55804.189 | 55734.276 | 0.138/0.375/0.487 |
| 4 | 52984.910 | 53184.150 | 53095.171 | 0.071/0.222/0.275/0.432 |
| 5 | 51983.825 | 52225.758 | 52117.712 | 0.048/0.124/0.150/0.338/0.341 |

*AIC* Akaike’s information criterion, *BIC* Bayesian information criterion, *SABIC* sample-adjusted Bayesian information criterion.

**Table S2** Risk of incident PAD for baseline TyG index among participants without any lipid- or glucose-lowering medication

| TyG index | Events/No. at risk | Model 1  HR (95% CI) | *P* value | Model 2  HR (95% CI) | *P* value | Model 3  HR (95% CI) | *P* value |
| --- | --- | --- | --- | --- | --- | --- | --- |
| Quartile 1 | 162/2396 | Reference | 1.0 | Reference | 1.0 | Reference | 1.0 |
| Quartile 2 | 213/2400 | 1.337 (1.090-1.641) | 0.005 | 1.204 (0.980-1.479) | 0.078 | 1.156 (0.939-1.422) | 0.172 |
| Quartile 3 | 254/2398 | 1.603 (1.314-1.956) | <0.001 | 1.327 (1.083-1.626) | 0.006 | 1.261 (1.026-1.551) | 0.028 |
| Quartile 4 | 283/2398 | 1.849 (1.518-2.252) | <0.001 | 1.370 (1.109-1.693) | 0.004 | 1.309 (1.054-1.624) | 0.015 |
| Per 1 SD (0.54) | 912/9592 | 1.239 (1.162-1.322) | <0.001 | 1.106 (1.028-1.190) | 0.007 | 1.091 (1.011-1.176) | 0.024 |

Model 1: Adjusted for baseline age, race, and sex.

Model 2: Adjusted for model 1 covariates plus baseline antihypertensive medication, body mass index, diabetes, drinking status, education level, leisure time index score, systolic blood pressure, smoking status, and sport index score.

Model 3: Adjusted for model 2 covariates plus baseline estimated glomerular filtration rate, factor VIII activity, fibrinogen, low-density lipoprotein cholesterol, von Willebrand factor, and white blood cell count.

*CI* confidence interval, *HR* hazard ratio, *PAD* peripheral artery disease, *TyG* triglyceride-glucose.

**Table S3** TyG index at examination visits by trajectory groups of TyG index

|  | TyG index trajectory groups | | | |
| --- | --- | --- | --- | --- |
|  | Low | Moderate | High | Very high |
| Visit 1 | 8.0±0.3 | 8.5±0.3 | 9.0±0.3 | 9.6±0.4 |
| Visit 2 | 8.1±0.3 | 8.6±0.3 | 9.1±0.3 | 9.8±0.4 |
| Visit 3 | 8.1±0.3 | 8.6±0.3 | 9.2±0.3 | 9.9±0.4 |
| Visit 4 | 8.2±0.3 | 8.7±0.3 | 9.2±0.4 | 9.9±0.5 |
| Visit 5 | 8.3±0.3 | 8.7±0.4 | 9.1±0.5 | 9.5±0.6 |
| Chang from Visit1 to Visit 5 | 0.06 (-0.02-0.14) | 0.05 (-0.04-0.14) | 0.03 (-0.07-0.14) | 0.05 (-0.11-0.18) |

*TyG* triglyceride-glucose.
